# Stress Response Mechanisms of *Salmonella* Enteritidis to Sodium Hypochlorite at the Proteomic Level

**DOI:** 10.3390/foods11182912

**Published:** 2022-09-19

**Authors:** Danhong Li, Shoukui He, Rui Dong, Yan Cui, Xianming Shi

**Affiliations:** MOST-USDA Joint Research Center for Food Safety, School of Agriculture and Biology, State Key Laboratory of Microbial Metabolism, Shanghai Jiao Tong University, Shanghai 200240, China

**Keywords:** foodborne pathogen, chlorine disinfectant, adaptation mechanism, comparative proteomics, bacterial physiology

## Abstract

*Salmonella* Enteritidis (*S.* Enteritidis) can adapt to sublethal sodium hypochlorite conditions, which subsequently triggers stress resistance mechanisms in this pathogen. Hence, the current work aimed to reveal the underlying stress adaptation mechanisms in *S.* Enteritidis by phenotypic, proteomic, and physiological analyses. It was found that 130 ppm sodium hypochlorite resulted in a moderate inhibitory effect on bacterial growth and an increased accumulation of intracellular reactive oxygen species. In response to this sublethal treatment, a total of 492 proteins in *S.* Enteritidis showed significant differential abundance (*p* < 0.05; fold change >2.0 or <0.5), including 225 more abundant proteins and 267 less abundant proteins, as revealed by the tandem-mass-tags-based quantitative proteomics technology. Functional characterization further revealed that proteins related to flagellar assembly, two-component system, and phosphotransferase system were in less abundance, while those associated with ABC transporters were generally in more abundance. Specifically, the repression of flagellar-assembly-related proteins led to diminished swimming motility, which served as a potential energy conservation strategy. Moreover, altered abundance of lipid-metabolism-related proteins resulted in reduced cell membrane fluidity, which provided a survival advantage to *S*. Enteritidis. Taken together, these results indicate that *S.* Enteritidis employs multiple adaptation pathways to cope with sodium hypochlorite stress.

## 1. Introduction

*Salmonella enterica* is a world-leading foodborne pathogen that is responsible for 93.8 million cases of gastroenteritis and 155,000 deaths each year [1]. Out of the >2610 serotypes, *Salmonella* Enteritidis (*S.* Enteritidis) stands out as the most common cause of human infections, which accounts for 40–60% salmonellosis [2,3]. The incidence of salmonellosis has been increasing in recent years, probably due to the ability of *S. enterica* to respond effectively to physical and chemical treatments commonly applied in food industries [4,5]. Hence, it is of paramount importance to uncover stress response mechanisms in this bacterium under food processing and storage-related conditions, such as chemical disinfection.

Sodium hypochlorite is a representative chlorine-based disinfectant that exerts sterilization activity by a one-electron transfer mechanism that attacks electron-rich cores in proteins and other biological molecules [6]. This disinfectant has been used in different concentrations to reduce microbial load in food industries (e.g., 50–200 ppm for food surfaces; 200–800 ppm on processing equipment) due to its easy operation, cost effectiveness, and excellent sterilization power [7,8]. Higher concentrations of sodium hypochlorite are generally avoided because its high oxidation power will lead to corrosion of the equipment. Moreover, sublethal concentrations of sodium hypochlorite can be encountered by foodborne pathogens as a consequence of improper storage, inappropriate utilization, or the presence of organic matter [9]. Adaptation of bacteria to sublethal sodium hypochlorite conditions can trigger stress resistance mechanisms in foodborne pathogens, such as *S. enterica* and *Escherichia coli* O157:H7 [8,10], which is a food safety concern. In this context, exploration of bacterial adaptation mechanisms to sodium hypochlorite has been a topic of great interest.

Primary biological approaches have been utilized to uncover stress response mechanisms of *S. enterica* to sublethal concentrations of sodium hypochlorite. DNA microarray analysis revealed that genes related to cysteine biosynthesis, energy metabolism, stress response, Fe-S cluster assembly, and biofilm formation were differentially expressed in *S.* Enteritidis and *Salmonella* Typhimurium under chlorine stress [8]. The role of several transcription factors (e.g., SlyA, MarA, and SoxS) in the response of *S.* Typhimurium to sodium hypochlorite was also characterized [11,12]. Nevertheless, it is currently unknown how *S.* Enteritidis responds to sodium hypochlorite at the proteomic level.

Analysis results by quantitative proteomics technology, such as tandem mass tags (TMT) and isobaric tags for relative and absolute quantification (iTRAQ), can provide key information on global physiological features of bacteria at the proteomic level [13]. This technique has been demonstrated to be powerful for the elucidation of stress response mechanisms in *S. enterica* to environmental stress factors (e.g., ethanol, heat, desiccation, acid, and ciprofloxacin) [14,15,16,17]. It is, therefore, expected that proteomic analysis will offer indispensable insight into the molecular bases of bacterial chlorine adaptation. Hence, the current work aimed to unravel stress adaptation mechanisms of *S.* Enteritidis to sodium hypochlorite based on TMT proteomics technology, which can provide potential protein targets for effective mitigation of chlorine resistance in this pathogen.

## 2. Materials and Methods

### 2.1. Bacterial Strains

*S*. Enteritidis ATCC 13076 was obtained from the American Type Culture Collection. This bacterium was conserved at −80 °C in LB (Luria-Bertani) broth containing 25% (*v*/*v*) glycerol in our laboratory. Prior to each experiment, the strain was thawed at 4 °C, streaked on the LB agar, and incubated overnight at 37 °C. A single colony was then inoculated into 5 mL LB broth, followed by overnight incubation at 37 °C/200 rpm to reach stationary phase for subsequent use.

### 2.2. Effect of Sodium Hypochlorite on Bacterial Survival Ability

An aliquot (1 mL) of stationary-phase bacterial suspension (approximately 10^9^ CFU/mL) prepared as described in Section 2.1 was added to 9 mL LB broth containing different amounts of sodium hypochlorite (Sigma, St. Louis, MN, USA). The final concentrations of sodium hypochlorite were 0, 65, 130, 260, and 520 ppm, respectively. The samples were then incubated at 37 °C/170 rpm for 6 h. The optical density at 600 nm (OD_600_) was measured at a one-hour interval by a microplate reader (Tecan Group Ltd., Männedorf, Switzerland). Each sample was run in triplicate from three biological replicates.

### 2.3. Quantification of Intracellular Reactive Oxygen Species (ROS)

The intracellular ROS levels of *S*. Enteritidis in the control (0 ppm sodium hypochlorite) and treated (130 ppm sodium hypochlorite) groups were quantified using a ROS assay kit (Beyotime Biotechnology, Shanghai, China) by the detection of 2′-7′-dichlorofluorescein fluorescence. *S*. Enteritidis cultures were collected by centrifugation and resuspended in 1 mL 2′,7′-dichlorodihydrofluorescein diacetate, followed by incubation at 37 °C for 20 min. The fluorometric assay was then conducted according to the manufacturer’s instructions. Each sample was run in triplicate from three biological replicates.

### 2.4. Protein Extraction, Quantification, and Digestion

*S*. Enteritidis cells in the control (0 ppm sodium hypochlorite) and treated (130 ppm sodium hypochlorite) groups from three biological replicates were washed three times with phosphate-buffered saline (PBS) and resuspended in 300 μL RIPA solution (250 mM Tris-HCl, pH 7.6; 150 mM NaCl; 10% Nonidet P-40; 10% sodium deoxycholate; 10% sodium dodecyl sulfate). After ultrasonicating for 2 min, the samples were centrifuged at 12,000 rpm for 10 min to obtain the supernatants. The protein concentrations in the supernatants were determined by Bicinchoninic acid (BCA) Protein Assay Reagent (Beyotime Biotechnology, Shanghai, China). A total of 100 μg protein from each sample was taken and diluted to about 1 mg/mL with distilled water. Five times volume of acetone was added to the sample and then precipitated overnight at −20 °C. The sample was then centrifuged at 12,000 rpm for 10 min at 4 °C. The precipitation was carefully obtained after being washed twice with 200 μL pre-chilled 80% acetone. Afterwards, 100 μL protein complex solutions were added and ultrasonicated for 5 min to solubilize protein precipitation. Afterwards, 5 mM DL-dithiothreitol was added and incubated for 10 min for the reduction of disulfide bonds. Proteins were alkylated with 10 mM iodoacetic acid in the dark for 15 min. Finally, 0.5 μg/μL of trypsin was mixed with samples and incubated overnight at 37 °C.

### 2.5. TMT Labeling and High-pH Pre-fractionation

TMT labeling was conducted according to the manufacturer’s instructions (Thermo Fisher Scientific, Rockford, IL, USA). Equal amounts of labeled samples from each group were mixed and trifluoroacetic acid was added to precipitate sodium deoxycholate. Labeled peptide samples were obtained after high-speed centrifugation for 10 min to collect the supernatant. The samples were then desalted using C18 Cartridges and concentrated by vacuum drying overnight at 4 °C. After lyophilization, the peptide samples were reconstituted to 50 μL with mobile phase A (10 mM ammonium acetate aqueous solution, pH 10) and separated under alkaline conditions using reversed-phase ultra-performance liquid chromatography (RP-UPLC). The gradient elution was conducted with 5% mobile phase B (10 mM ammonium acetate, 10% H_2_O, 90% acetonitrile, pH 10) for 2 min, 5–30% mobile phase B for 40 min, 30–40% mobile phase B for 10 min, 40–90% mobile phase B for 4 min, 90% mobile phase B for 2 min, and 2% mobile phase B for 2 min. The fractions were collected every minute and then stored at −80 °C after vacuum drying.

### 2.6. Nano-Liquid Chromatography-Mass Spectrometry/Mass Spectrometry Analysis

About 1 μg of total peptides were separated by a nanoUPLC liquid system EASYnLC1200 coupled with a QExactive HFX (Thermo Fisher Scientific) for data collection. A reversed-phase chromatographic column (100 μm ID × 15 cm, Reprosil Pur 120 C18AQ, 1.9 μm, Dr. Math) was used in the chromatographic separation. After the chromatographic column was equilibrated with 100% phase A (0.1% formic acid-98% aqueous solution), the sample was directly loaded onto the chromatographic column by the autosampler and then separated by the chromatographic column gradient with 300 nL/min of flow rate and 90 min of gradient duration. The gradient elution was conducted with 2–5% mobile phase B (0.1% formic acid-80% acetonitrile solution) for 2 min, 5–22% mobile phase B for 68 min, 22–45% mobile phase B for 16 min, 45–95% mobile phase B for 2 min, and 95% mobile phase B for 2 min.

Data dependent acquisition (DDA) mode was used in mass spectrometry (MS) analysis and positive ion detection mode with a total analysis time of 90 min. For MS-1, the resolution was 120,000 and the *m*/*z* range was 350–1600; for MS-2, the resolution was 45,000 and a fixed first mass was 110. The automatic gain control target for MS-1 was set to 3E6 with max IT 30 ms, and 1E5 for MS-2 with max IT 96 ms. The 20 ions with the highest intensity in the primary scan were screened by the quadrupole and fragmented by higher-energy collisional dissociation with normalized collision energy of 32% and isolation window of 0.7 *m*/*z*. The dynamic exclusion time was set to 45 s. Single-charge and >6 valence ions were excluded from the DDA procedure.

### 2.7. Peptide Analysis and Protein Identification

Raw MS processing and peptide identification were performed with Proteome Discoverer 2.4.0.305 and Mascot Server 2.6.1. MS data were searched against the *S*. Enteritidis UniProt FASTA database. Precursor ion mass tolerance and fragment ion tolerance were set to 10 ppm and 0.02 Da, respectively. Tryptic peptides with a maximum of 2 missed cleavages were allowed. The false discovery rate was 1% for peptide spectrum matches and peptide levels. Unique peptide and Razor peptide were utilized for protein quantification and total peptide amount for normalization.

### 2.8. Bioinformatics Analysis

The Gene Ontology (GO) program was utilized to annotate differentially abundant proteins in terms of cell component, biological process, and molecular function. KEGG (Kyoto Encyclopedia of Genes and Genomes) pathway enrichment analysis was employed to conduct pathway analysis of differentially abundant proteins.

### 2.9. Gene Expression Analysis

Several differently abundant proteins in the TMT test were used to analyze the corresponding transcriptional profiles by the reverse transcription-quantitative real-time PCR (RT-qPCR). The primers in Table 1 were designed via NCBI-Nucleotide BLAST in the current work. The total RNA was extracted from control (0 ppm sodium hypochlorite) and treated (130 ppm sodium hypochlorite) groups using the Trizol reagent according to the manufacturer’s protocol. After extraction, the concentration of RNA and the RNA integrity were determined by a Nanodrop 2000c spectrophotometer and 1% agarose gel electrophoresis. DNA removal and reverse transcription were conducted using the PrimeScript RT reagent kit with gDNA Eraser (Takara, Dalian, China). The removal effect of DNA was validated by PCR and 1% agarose gel electrophoresis. The resulting cDNA served as the template for real-time PCR using SYBR Premix Ex Taq II reagents (Takara, Dalian, China). The 16S rRNA gene was chosen as the reference gene [18]. The program of RT-qPCR was listed as follows: 95 °C/3 min; 95 °C/10 s, 60 °C/15 s, 68 °C/20 s, 40 cycles; 95 °C/15 s; 60 °C/1 min; 95 °C/15 s. Melting curve and amplification efficiency were performed to validate the primer specificity. Each sample was run in duplicate from three biological replicates. The relative gene expression was calculated using the 2^−^^△△CT^ method [14]. Gene expression was considered as significant (*p* < 0.05) when the relative expression level was over two-fold.

### 2.10. Membrane Fatty Acid Composition Analysis

*S*. Enteritidis cells in the control (0 ppm sodium hypochlorite) and treated (130 ppm sodium hypochlorite) groups were spun at 8000 rpm for 2 min and washed three times with PBS. Fatty acid methyl esters were extracted from 40–60 mg pelleted cells, followed by a gas-chromatography analysis as detailed in our previous work [19]. The relative level of each fatty acid was presented as its molar ratio to the total fatty acids. Each sample was run in duplicate from three biological replicates.

### 2.11. Swimming Motility Assay

Flagellar motility of *S*. Enteritidis in the control (0 ppm sodium hypochlorite) and treated (130 ppm sodium hypochlorite) groups was quantified by a soft agar (0.3%) test as previously described [20]. LB broth enriched with 0.3% agar was autoclaved and allowed to dry at room temperature. An aliquot of 2 μL bacterial suspension was then inoculated by piercing the surface of fresh agar. Bacterial motility was recorded after the plates were incubated at 37 °C for 12 h. Each sample was run in triplicate from five biological replicates.

### 2.12. Statistical Analysis

Proteomic, gene expression, and physiological data in the treated group (130 ppm sodium hypochlorite) were compared with those in the control group (0 ppm sodium hypochlorite). To determine if a protein was differentially abundant in response to sodium hypochlorite, the fold change >2.0 or <0.5 and *t*-test *p*-value < 0.05 were applied as the selection standard. In other cases, statistical analyses were conducted by one-way ANOVA followed by Duncan’s tests at the level of *p* < 0.05 using SPSS Software.

## 3. Results and Discussion

### 3.1. Selection for the Sublethal Concentration of Sodium Hypochlorite to Treat S. Enteritidis

**Figure 1:**
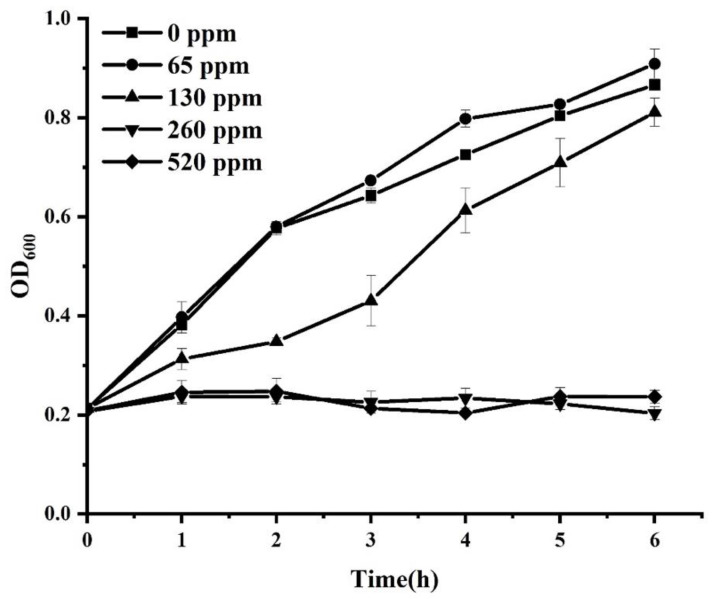
Growth curve of *S*. Enteritidis in different concentrations of sodium hypochlorite.

The influence of sodium hypochlorite on the growth of *S*. Enteritidis was initially determined. As shown in Figure 1, sodium hypochlorite at the concentration of 65 ppm did not significantly (*p* > 0.05) affect the growth of *S*. Enteritidis. However, *S*. Enteritidis treated with 130, 260, and 520 ppm sodium hypochlorite exhibited significantly (*p* < 0.05) reduced growth compared with the control group. A moderate inhibitory effect on *S*. Enteritidis was observed in the presence of 130 ppm sodium hypochlorite, while complete growth inhibition was achieved when chlorine concentration was increased to 260 or 520 ppm. Chemical agents at sublethal concentrations have been considered to exert an inhibitory but not lethal effect on bacterial pathogens [20]. Therefore, 130 ppm sodium hypochlorite was selected for sublethal adaptation treatments in the subsequent test.

### 3.2. ROS Production in S. Enteritidis in Response to Sodium Hypochlorite

**Figure 2:**
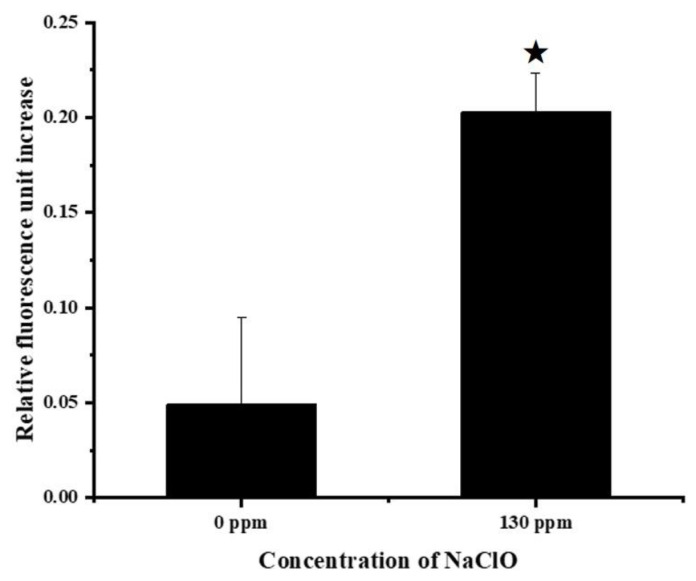
Changes in ROS generation in *S*. Enteritidis in response to 130 ppm sodium hypochlorite. Asterisk indicates a significant difference (*p* < 0.05).

Sodium hypochlorite may trigger a Fenton-type reaction and generate hydroxyl radicals, which have adverse effects on bacterial growth [8]. Hence, the ROS level in *S*. Enteritidis was measured after sublethal sodium hypochlorite adaptation treatments. As shown in Figure 2, exposure to sodium hypochlorite at 130 ppm for 2 h resulted in an increase in the intracellular ROS levels, which represented the induction of oxidative stress. The production of ROS can account for the inhibitory effect of sublethal sodium hypochlorite adaptation on *S*. Enteritidis growth. Nevertheless, it is currently unknown how *S*. Enteritidis responds to oxidative stress posed by sodium hypochlorite at the proteomic level. Therefore, a TMT isobaric labeling technique was utilized to uncover the strategies employed by this bacterium to survive under sodium hypochlorite stress.

### 3.3. Overview of Quantitative Proteomics Results

Global proteomic profiling of *S*. Enteritidis in response to 130 ppm sodium hypochlorite was determined by the TMT isobaric labeling technique to explore the underlying adaptation mechanisms. A total of 35,096 peptides and 3786 proteins were identified in three independent trials. According to the commonly used cutoff criteria (*p* < 0.05; fold change >2.0 or <0.5) [21], a total of 492 differentially abundant proteins were identified, with 225 in more abundance and 267 in less abundance. Similarly, *S*. Enteritidis altered the abundance of hundreds of proteins to respond to ethanol disinfectant and hydrochloric acid [14,15].

### 3.4. Comparision between Proteomics Data and Gene Expression Profiles

**Figure 3:**
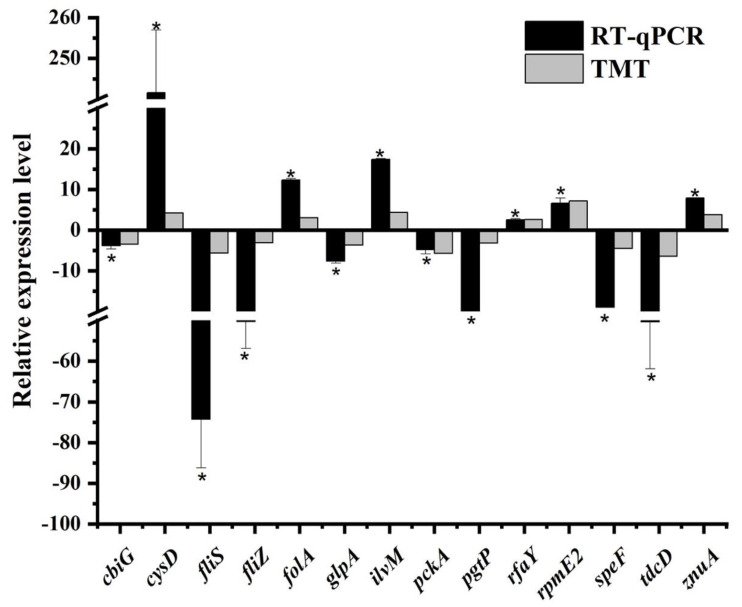
Comparison between protein abundance patterns and gene expression profiles in *S*. Enteritidis in response to 130 ppm sodium hypochlorite. Asterisk indicates a significant difference (*p* < 0.05).

The mRNA expression levels of 14 genes were determined via RT-qPCR analysis in the current work. These genes were responsible for encoding 14 differentially abundant proteins revealed by the TMT-based proteomic analysis, of which eight (CbiG, FliS, FliZ, GlpA, PckA, PgtP, SpeF, and TdcD) were decreased in abundance and six (CysD, FolA, IlvM, RfaY, RpmE2, and ZnuA) were increased in abundance. As depicted in Figure 3, the trend in gene expression and protein abundance under sodium hypochlorite stress was generally similar. This provided evidence for the feasibility of using the proteomic approach to elucidate stress response mechanisms of *S*. Enteritidis [13,14].

### 3.5. Functional Characterization of Differentially Abundant Proteins

**Figure 4:**
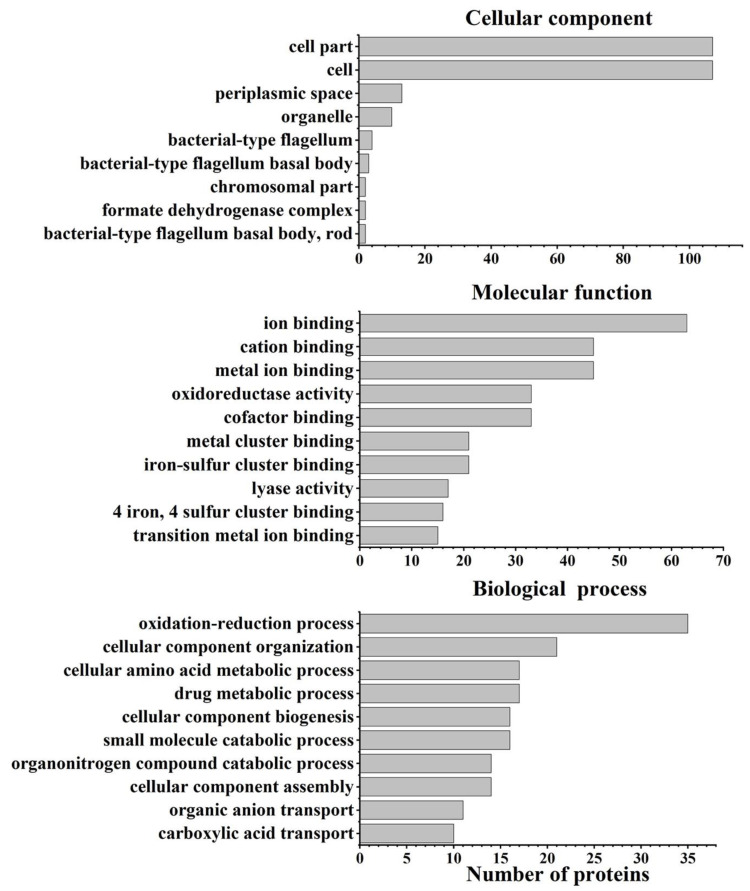
GO enrichment analysis of differentially abundant proteins in *S*. Enteritidis in response to 130 ppm sodium hypochlorite.

Bioinformatics analysis was conducted on all differentially abundant proteins to uncover the survival mechanisms of *S*. Enteritidis under oxidative stress produced by 130 ppm sodium hypochlorite. GO characterization was initially carried out to explore the biological process, cellular component, and molecular function (Figure 4). Biological process characterization indicated that these proteins were related to the oxidation–reduction process, cellular component assembly, organonitrogen compound catabolic process, etc. Cellular component analysis suggested that these proteins were located in cells, cell parts, periplasmic spaces, etc. In terms of molecular function, these proteins were involved in metal ion binding, cofactor binding, oxidoreductase activity, etc.

**Figure 5:**
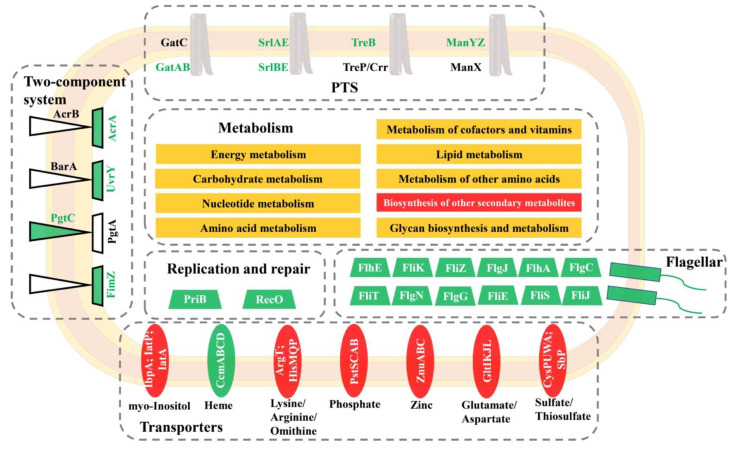
A proposed model for the adaptation of *S*. Enteritidis to 130 ppm sodium hypochlorite. Red indicates that a certain pathway contained proteins with increased abundance, green indicates that a certain pathway contained proteins with decreased abundance, and yellow indicates that a certain pathway contained proteins with increased and decreased abundance.

KEGG pathway analysis indicated that differentially abundant proteins were significantly enriched in cellular metabolism, two-component system, ABC transporter, phosphotransferase system, and flagellar assembly. A proposed model was further formulated for these proteomic changes (Figure 5), highlighting that a complex network regulated the response of *S*. Enteritidis to sodium hypochlorite. Similarly, *S*. Enteritidis also employed multiple pathways to cope with other stress factors, such as ethanol, acid, and egg white [22,23,24].

#### 3.5.1. Cellular Metabolism

**Figure 6:**
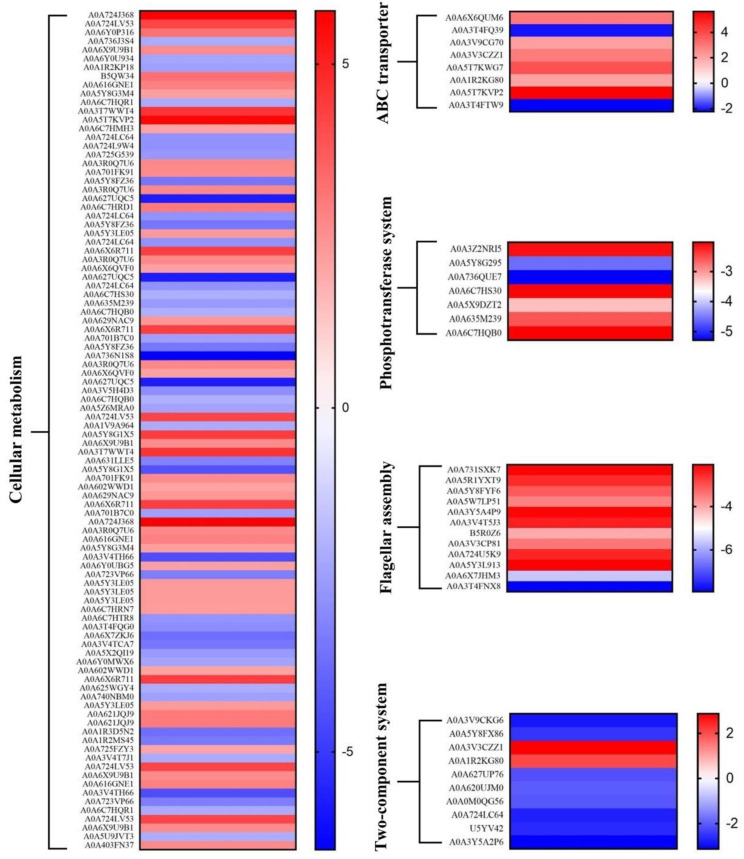
Heat map of differentially abundant proteins in *S*. Enteritidis during adaptation to 130 ppm sodium hypochlorite.

A considerable number of proteins related to cellular metabolism were differentially abundant in *S*. Enteritidis during the course of adaptation with sodium hypochlorite (Figure 6). These proteins belonged to amino acid, nucleotide, energy, carbohydrate, cofactors and vitamins, lipid, other amino acids, other secondary metabolites, and glycan metabolic pathways. This indicated a coordinated regulation of different bacterial metabolic processes in response to sodium hypochlorite commonly used in food industries. Similarly, genes involved in amino acid and nucleotide biosynthesis were differentially expressed after exposure of *Salmonella* Newport to sodium hypochlorite [25], highlighting bacterial requirements for nutrients and DNA repair to adapt to sodium hypochlorite treatments.

In the lipid metabolism pathway, most proteins associated with the glycerophospholipid metabolism (e.g., GlpA, GlpC) and unsaturated fatty acid biosynthesis (e.g., YciA) were decreased in abundance in the current work. In particular, regulation of fatty acid composition was closely related to bacterial stress responses [19]. Therefore, the role of fatty acid biosynthesis in the response of *S*. Enteritidis to sodium hypochlorite merits further investigation.

#### 3.5.2. Two-Component System

In the current work, two-component systems, including BarA-UvrY, PgtAB, and ArcAB, were repressed in response to the sodium hypochlorite treatment in *S*. Enteritidis. A two-component system consists of a transmembrane histidine kinase and a cytoplasmic response regulator, which plays a crucial role in regulating bacterial stress responses [26]. BarA-UvrY is an important global regulator of virulence, motility, and biofilm formation by influencing the expression of sRNAs in *S*. Typhimurium [27]. PgtAB controls a phosphoglycerate transport system in *S. enterica*, which is encoded by the *pgtABCP* operon [28]. Therefore, the contribution of these two-component systems to sodium hypochlorite resistance in *S*. Enteritidis will be a focus of our future research.

In particular, ArcAB is a multi-drug exclusion system that is important in the resistance of bacteria to ROS [28]; deletion of AcrAB made *E. coli* mutants more sensitive to disinfectants, antibiotics, and dyes [29,30,31]. Interestingly, the AcrAB system was decreased in abundance in *S*. Enteritidis with moderate growth in the presence of sodium hypochlorite in spite of the higher ROS production in the current work. Thus, we infer that there must exist other mechanisms involved in the response of *S*. Enteritidis to chlorine stress.

#### 3.5.3. ABC Transporter

A couple of proteins related to ABC transporters were differently abundant in the current study, indicating their involvement in sodium hypochlorite adaptation in *S*. Enteritidis (Figure 6). Briefly, proteins responsible for transport of myo-Inositol (IbpA), arginine (ArgT), phosphate (PstS), zinc (ZnuA), glutamate (GltL), and sulfate/thiosulfate (CysW) were increased in abundance, while those for heme (CcmA and CcmB) were decreased in abundance. There was only one transporter, CcmABCD, with more than one differently abundant protein in response to sodium hypochlorite treatments.

CcmABCD is a transporter that facilitates the export of heme into the periplasm for cytochrome biosynthesis [30,31]. Heme is a cofactor for some cytochromes and is required for bacterial defense against oxygen intermediates, such as hydrogen peroxide [32]. This indicated the crucial role of CcmABCD transporter in sodium hypochlorite resistance of *S*. Enteritidis. Similarly, the expression of this heme transporter was also altered in ceftiofur-tolerant strains of *S*. Enteritidis, which might help to minimize ceftiofur concentrations in the periplasm and thus conferred bacterial ceftiofur resistance [31].

#### 3.5.4. Phosphotransferase System

As shown in Figure 6, less abundant proteins were also observed in the phosphotransferase system (PTS) in response to sodium hypochlorite treatments in *S*. Enteritidis. These proteins included PTS mannose/fructose/sorbose transporter ManY, PTS galactitol transporter subunit GatB, PTS trehalose transporter subunit TreB, etc. PTS is required in the process of glycolysis, which is integral for *Salmonella* infection in host cells [33]. Therefore, the repression of PTS-related proteins might result in a reduction in pathogenic potential of *S*. Enteritidis.

It should be noted that the aforementioned PTS proteins were involved in bacterial response to diverse stresses. For instance, the *manXYZ* operon was downregulated under chlorine treatments in *S. enterica* [8]; overexpression of this operon contributed to organic solvent tolerance of *E. coli* [34]. The expression of *gatA* and *gatB* genes in *E. coli* was activated in response to superoxide stress generated by paraquat [35]. Moreover, a two-fold increase in the expression level of *treB* occurred in *Salmonella* Tennessee after desiccation [36]. These results enforced the knowledge of PTS playing an important role in bacterial stress responses.

#### 3.5.5. Flagellar Assembly

In the current study, adaptation to sodium hypochlorite resulted in the repression of a large number of proteins responsible for flagella assembly in *S*. Enteritidis, including flagellar basal-body rod protein FlgCJNG (accession no. A0A3V4T5J3; A0A5W7LP51; A0A3V3CP81; A0A724U5K9) and flagella biosynthesis regulatory protein FliZ (accession no. A0A5Y8FYF6) (Figure 6). A previous transcriptomic analysis demonstrated that flagellar basal body genes (e.g., *flgCG*) and flagellar biosynthesis genes (e.g., *fliA*, *fijB*) were also downregulated in response to chlorine treatments in the same bacterium [8]. Moreover, the expression of flagellar-related genes in *S. enterica* was reduced in the presence of many other food processing and storage-related stresses, such as ethanol, acid, heat, and desiccation [14,37,38,39]. Therefore, inhibition of flagellar assembly seemed to be a universal stress response mechanism in *S. enterica*, which might be an energy conservation strategy for bacteria to cope with sodium hypochlorite stress.

### 3.6. Fatty Acid Composition in S. Enteritidis in Response to Sodium Hypochlorite

In the current study, several proteins related to lipid metabolism were differentially abundant, of which YciA (accession no. A0A3V4T7J1) involved in unsaturated fatty acid biosynthesis was repressed. Thus, fatty acid compositions were further analyzed, and the results are displayed in Table 2. The content of the majority of unsaturated fatty acids (e.g., C16:1n6 and C18:1n9c) was significantly (*p* < 0.05) decreased, while that of most saturated fatty acids (e.g., C12:0 and C15:0) was significantly (*p* < 0.05) increased. As a result, a significantly (*p* < 0.05) lower ratio of total unsaturated/saturated fatty acids was observed in bacterial cells adapted with sodium hypochlorite, indicating a reduction in cell membrane fluidity.

Regulation of cell membrane fluidity via fatty acid composition has been suggested as a crucial mechanism for bacterial stress responses [40]. For example, acid adaptation reduced the membrane fluidity of *S*. Enteritidis, thus increasing bacterial resistance to acid and heat treatments [41]. It was thus indicative that the repression of most proteins in the lipid metabolism pathway potentially resulted in reduced cell membrane fluidity, which subsequently provided a survival advantage to *S*. Enteritidis in the presence of sodium hypochlorite in the current work.

### 3.7. Flagellar Motility in S. Enteritidis in Response to Sodium Hypochlorite

**Figure 7:**
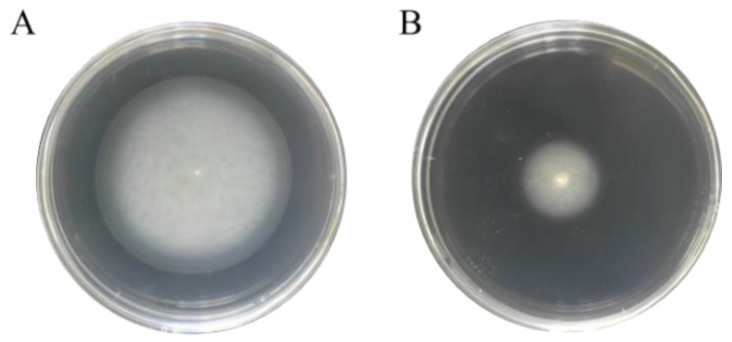
Effect of sodium hypochlorite on swimming motility of *S*. Enteritidis. (**A**) Control group (0 ppm sodium hypochlorite); (**B**) treated group (130 ppm sodium hypochlorite).

*S*. Enteritidis proteins related to flagella assembly were produced in less abundance in response to 130 ppm sodium hypochlorite in the current study. Therefore, a swimming assay was further carried out using 0.3% soft agar to reveal whether this protein expression alteration could affect bacterial flagellar motility. As depicted in Figure 7, *S*. Enteritidis formed a large halo migrating from the center almost to the edge of the agar after 12 h of incubation in the absence of sodium hypochlorite. On the contrary, sodium hypochlorite remarkedly abrogated this migration and only a small halo was observed. It was thus indicative that flagellar motility of *S*. Enteritidis was retarded by sodium hypochlorite.

In line with the above-mentioned findings, sublethal levels of ethanol were also able to inhibit swimming motility of *S*. Enteritidis [20]. Furthermore, flagellar motility of *S*. Typhimurium was completely abolished by agaric acid [42]. Interestingly, our current study enforced the knowledge of the repression of flagellar-assembly-related proteins leading to reduced cell motility of *S*. Enteritidis under sublethal sodium hypochlorite stress. This phenomenon has been considered as an energy conservation strategy for bacteria to survive in stressful environments [23].

## 4. Conclusions

*S*. Enteritidis utilized hundreds of proteins involved in multiple pathways, including cellular metabolism, two-component system, ABC transporter, phosphotransferase system, and flagellar assembly to survive under sublethal sodium hypochlorite stress. Of note, lipid-metabolism-mediated cell membrane fluidity and flagellar-assembly-regulated cell motility offered a survival advantage to *S*. Enteritidis. Future research can be focused on characterization of other metabolic pathways to further uncover regulatory networks coordinating bacterial chlorine adaptation and potential therapeutic targets for *S*. Enteritidis. Such knowledge will contribute to the formulation of more effective intervention strategies to ensure food safety.

## Figures and Tables

**Table 1:** Primers used in the gene expression test.

Gene	Sequence (5′ to 3′)
16S rRNA	F: CAGAAGAAGCACCGGCTAAC
	R: GACTCAAGCCTGCCAGTTTC
*cbiG*	F: TCCCGTACTGCACTGGAAAC
	R: CTTTTAACGCCAGCGGATCG
*cysD*	F: TCAATCCGTTCGTTCACGGT
	R: GGATTTTTCCTCATCGCGCC
*fliS*	F: CGATGTTGTCGCGAAAGGTG
	R: CGCAATCTCACCGCCTTTTT
*fliZ*	F: TGCAGGACGGTTTTCTCGAT
	R: CTGGCGGTAAAGGGGGATTT
*folA*	F: CGATGCGCCGGAAATTATGG
	R: CCGGAAAATGGGTATCGCCT
*glpA*	F: CATGTCATTAACCGCTGCCG
	R: TCGACTTCATCTGCGGTGAC
*ilvM*	F: CGGTCGGTCGACTTACTGTT
	R: TTTGTTGTGATGTGGCAGCG
*pckA*	F: ACGCAGTATGCTGAAGTGCT
	R: TTGCGCGCGTATCTTTGATG
*pgtP*	F: TGGTACTCTGCGCGATTGTT
	R: ATTGAAGACCACCAGAGCGG
*rfaY*	F: AGAAGGGTTTACGGCGCTTA
	R: TCCCTCTCACCTCGTCAGAA
*rpmE2*	F: CGACACCAGCGCAAATGAAT
	R: GATATGTCACGCCGTCCAGT
*speF*	F: ATTGATGGTAAGCCGTGGCA
	R: TGTTCTCCCGGAACGAAGTG
*tdcD*	F: GAAAAAGCCTGGCACGAAGG
	R: CCATCCAGCCGATGTAACGA
*znuA*	F: CATTGCTGATGGCGTTACGG
	R: CGCCCTGTAAGCGTTTTACG

**Table 2:** Effect of sodium hypochlorite on fatty acid composition of *S*. Enteritidis.

Fatty Acid	Control Group	Treated Group
C12:0	1.99 ± 0.31	6.25 ± 2.39 *
C14:0	4.94 ± 0.20	2.56 ± 0.68
C14:1n5	0.45 ± 0.13	3.46 ± 2.17
C15:0	18.03 ± 3.65	32.80 ± 2.08 *
C16:0	36.89 ± 0.23	34.40 ± 7.63
C16:1n6	10.90 ± 1.67	3.61 ± 0.46 *
C17:0	1.07 ± 0.59	2.69 ± 0.37
C17:1n7	3.94 ± 1.49	0.99 ± 0.56
C18:0	7.70 ± 1.80	11.64 ± 3.15
C18:1n9c	14.11 ± 2.42	1.61 ± 1.95 *
UFA/SFA ratio	0.39 ± 0.04	0.09 ± 0.03 *

Note: asterisk indicates a significant difference (*p* < 0.05) in the fatty acid level between treated group (130 ppm sodium hypochlorite) and control group (0 ppm sodium hypochlorite). UFA, unsaturated fatty acids; SFA, saturated fatty acids.

## Data Availability

The data are available upon reasonable request.

## References

[1] Moe A.Z., Paulsen P., Pichpol D., Fries R., Irsigler H., Baumann M.P.O., Oo K.N. (2017). Prevalence and antimicrobial resistance of *Salmonella* isolates from chicken carcasses in retail markets in Yangon, Myanmar. J. Food Prot..

[2] Mezal E.H., Sabol A., Khan M.A., Ali N., Stefanova R., Khan A.A. (2014). Isolation and molecular characterization of *Salmonella enterica* serovar Enteritidis from poultry house and clinical samples during 2010. Food Microbiol..

[3] Pearce M.E., Alikhan N.F., Dallman T.J., Zhou Z., Grant K., Maiden M.C.J. (2018). Comparative analysis of core genome MLST and SNP typing within a European *Salmonella* serovar Enteritidis outbreak. Int. J. Food Microbiol..

[4] Humphrey T. (2004). *Salmonella*, stress responses and food safety. Nat. Rev. Microbiol..

[5] Ricke S.C., Dawoud T.M., Kim S.A., Park S.H., Kwon Y.M. (2018). *Salmonella* cold stress response: Mechanisms and occurrence in foods. Adv. Appl. Microbiol..

[6] Byun K.H., Han S.H., Yoon J., Park S.H., Ha S.D. (2021). Efficacy of chlorine-based disinfectants (sodium hypochlorite and chlorine dioxide) on *Salmonella* Enteritidis planktonic cells, biofilms on food contact surfaces and chicken skin. Food Control.

[7] Van Houdt R., Michiels C.W. (2010). Biofilm formation and the food industry, a focus on the bacterial outer surface: Biofilm formation and the bacterial outer surface. J. Appl. Microbiol..

[8] Wang S., Phillippy A.M., Deng K., Rui X., Li Z., Tortorello M.L., Zhang W. (2010). Transcriptomic responses of *Salmonella enterica* serovars Enteritidis and Typhimurium to chlorine-based oxidative stress. Appl. Environ. Microbiol..

[9] Capita R., Buzón-Durán L., Riesco-Peláez F., Alonso-Calleja C. (2017). Effect of sub-lethal concentrations of biocides on the structural parameters and viability of the biofilms formed by *Salmonella* Typhimurium. Foodborne Pathog. Dis..

[10] Wang S., Deng K., Zaremba S., Deng X., Lin C., Wang Q., Tortorello M.L., Zhang W. (2009). Transcriptomic response of *Escherichia coli* O157:H7 to oxidative stress. Appl. Environ. Microbiol..

[11] Cabezas C.E., Briones A.C., Aguirre C., Pardo-Esté C., Castro-Severyn J., Salinas C.R., Baquedano M.S., Hidalgo A.A., Fuentes J.A., Morales E.H. (2018). The transcription factor SlyA from *Salmonella* Typhimurium regulates genes in response to hydrogen peroxide and sodium hypochlorite. Res. Microbiol..

[12] Collao B., Morales E.H., Gil F., Polanco R., Calderón I.L., Saavedra C.P. (2012). Differential expression of the transcription factors MarA, Rob, and SoxS of *Salmonella* Typhimurium in response to sodium hypochlorite: Down-regulation of *rob* by MarA and SoxS. Arch. Microbiol..

[13] Arunima A., Yelamanchi S.D., Padhi C., Jaiswal S., Ryan D., Gupta B., Sathe G., Advani J., Gowda H., Prasad T.S.K. (2017). “Omics” of food-borne gastroenteritis: Global proteomic and mutagenic analysis of *Salmonella enterica* serovar Enteritidis. OMICS.

[14] He S., Qin X., Wong C.W.Y., Shi C., Wang S., Shi X. (2019). Ethanol adaptation strategies in *Salmonella enterica* serovar Enteritidis revealed by global proteomic and mutagenic analyses. Appl. Environ. Microbiol..

[15] Hu S., Yu Y., Lv Z., Shen J., Ke Y., Xiao X. (2020). Proteomics study unveils ROS balance in acid-adapted *Salmonella* Enteritidis. Food Microbiol..

[16] Li L., Wang W., Zhang R., Xu J., Wang R., Wang L., Zhao X., Li J. (2018). First acetyl-proteome proﬁling of *Salmonella* Typhimurium revealed involvement of lysine acetylation in drug resistance. Vet. Microbiol..

[17] Maserati A., Lourenco A., Diez-Gonzalez F., Fink R.C. (2018). iTRAQ-based global proteomic analysis of *Salmonella enterica* serovar Typhimurium in response to desiccation, low water activity, and thermal treatment. Appl. Environ. Microbiol..

[18] He S., Cui Y., Qin X., Zhang F., Shi C., Paoli G.C., Shi X. (2018). Influence of ethanol adaptation on *Salmonella enterica* serovar Enteritidis survival in acidic environments and expression of acid tolerance-related genes. Food Microbiol..

[19] Qin X., Dong R., He S., Zhou X., Zhang Z., Cui Y., Shi X. (2020). Characterization of the role of *ybgC* in lysozyme resistance of *Salmonella* Enteritidis. Food Control.

[20] He S., Zhan Z., Shi C., Wang S., Shi X. (2022). Ethanol at subinhibitory concentrations enhances biofilm formation in *Salmonella* Enteritidis. Foods.

[21] Zhong Q., Tian J., Wang J., Fang X., Liao Z. (2018). iTRAQ-based proteomic analysis of the viable but nonculturable state of *Vibrio parahaemolyticus* ATCC 17802 induced by food preservative and low temperature. Food Control.

[22] Baron F., Bonnassie S., Alabdeh M., Cochet M.F., Nau F., Guérin-Dubiard C., Gautier M., Andrews S.C., Jan S. (2017). Global gene-expression analysis of the response of *Salmonella* Enteritidis to egg white exposure reveals multiple egg white-imposed stress responses. Front. Microbiol..

[23] He S., Cui Y., Dong R., Chang J., Cai H., Liu H., Shi X. (2022). Global transcriptomic analysis of ethanol tolerance response in *Salmonella* Enteritidis. Curr. Res. Food Sci..

[24] Hu S., Yu Y., Zhou D., Li R., Xiao X., Wu H. (2018). Global transcriptomic acid tolerance response in *Salmonella* Enteritidis. LWT-Food Sci. Technol..

[25] Dunn L.L., Smith D.M., Critzer F.J. (2020). Transcriptomic behavior of *Salmonella enterica* Newport in response to oxidative sanitizers. J. Food Prot..

[26] Murret-Labarthe C., Kerhoas M., Dufresne K., Daigle F. (2020). New roles for two-component system response regulators of *Salmonella enterica* serovar Typhi during host cell interactions. Microorganisms.

[27] Teplitski M., Al-Agely A., Ahmer B.M.M. (2006). Contribution of the SirA regulon to biofilm formation in *Salmonella enterica* serovar Typhimurium. Microbiology.

[28] de Pina L.C., da Silva F.S.H., Galvão T.C., Pauer H., Ferreira R.B.R., Antunes L.C.M. (2021). The role of two-component regulatory systems in environmental sensing and virulence in *Salmonella*. Crit. Rev. Microbiol..

[29] Cha H., Pos K.M. (2014). Cooperative transport mechanism and proton-coupling in the multidrug efflux transporter complex ArcAB-TolC. Membrane Transport Mechanism: 3D Structure and Beyond.

[30] Christensen O., Harvat E.M., Thöny-Meyer L., Ferguson S.J., Stevens J.M. (2007). Loss of ATP hydrolysis activity by CcmAB results in loss of *c*-type cytochrome synthesis and incomplete processing of CcmE. FEBS J..

[31] Radford D., Strange P., Lepp D., Hernandez M., Rehman M.A., Diarra M.S., Balamurugan S. (2018). Genomic and proteomic analyses of *Salmonella enterica* serovar Enteritidis identifying mechanisms of induced *de novo* tolerance to ceftiofur. Front. Microbiol..

[32] Elgrably-Weiss M., Park S., Schlosser-Silverman E., Rosenshine I., Imlay J., Altuvia S.A. (2002). *Salmonella enterica* serovar Typhimurium *hema* mutant is highly susceptible to oxidative DNA damage. J. Bacteriol..

[33] Bowden S.D., Rowley G., Hinton J.C.D., Thompson A. (2009). Glucose and glycolysis are required for the successful infection of macrophages and mice by *Salmonella enterica* serovar Typhimurium. Infect. Immun..

[34] Okochi M., Kurimoto M., Shimizu K., Honda H. (2007). Increase of organic solvent tolerance by overexpression of *manXYZ* in *Escherichia coli*. Appl. Microbiol. Biotechnol..

[35] Pomposiello P.J., Bennik M.H.J., Demple B. (2001). Genome-wide transcriptional profiling of the *Escherichia coli* responses to superoxide stress and sodium salicylate. J. Bacteriol..

[36] Li H., Bhaskara A., Megalis C., Tortorello M.L. (2012). Transcriptomic analysis of *Salmonella* desiccation resistance. Foodborne Pathog. Dis..

[37] Cui X., Hu C., Ou L., Kuramitsu Y., Masuda Y., Honjoh K., Miyamoto T. (2019). Transcriptional analysis on heat resistance and recovery from thermal damage in *Salmonella* under high salt condition. LWT-Food Sci. Technol..

[38] Finn S., Händler K., Condell O., Colgan A., Cooney S., McClure P., Amézquita A., Hinton J.C.D., Fanning S. (2013). Prop is required for the survival of desiccated *Salmonella enterica* serovar Typhimurium cells on a stainless steel surface. Appl. Environ. Microbiol..

[39] Ryan D., Pati N.B., Ojha U.K., Padhi C., Ray S., Jaiswal S., Singh G.P., Mannala G.K., Schultze T., Chakraborty T. (2015). Global transcriptome and mutagenic analyses of the acid tolerance response of *Salmonella enterica* serovar Typhimurium. Appl. Environ. Microbiol..

[40] Yoon Y., Lee H., Lee S., Kim S., Choi K.H. (2015). Membrane fluidity-related adaptive response mechanisms of foodborne bacterial pathogens under environmental stresses. Food Res. Int..

[41] Yang Y., Kadim M.I., Khoo W.J., Zheng Q., Setyawati M.I., Shin Y.J., Yuk H.G. (2014). Membrane lipid composition and stress/virulence related gene expression of *Salmonella* Enteritidis cells adapted to lactic acid and trisodium phosphate and their resistance to lethal heat and acid stress. Int. J. Food Microbiol..

[42] Lories B., Belpaire T.E., Yssel A., Ramon H., Steenackers H.P. (2020). Agaric acid reduces *Salmonella* biofilm formation by inhibiting flagellar motility. Biofilm.

